# Safety and efficacy of combination therapy of interferon‐α2 and ruxolitinib in polycythemia vera and myelofibrosis

**DOI:** 10.1002/cam4.1619

**Published:** 2018-06-22

**Authors:** Stine Ulrik Mikkelsen, Lasse Kjær, Mads Emil Bjørn, Trine Alma Knudsen, Anders Lindholm Sørensen, Christen Bertel Lykkegaard Andersen, Ole Weis Bjerrum, Nana Brochmann, Daniel El Fassi, Torben A. Kruse, Thomas Stauffer Larsen, Hans Torben Mourits‐Andersen, Claus Henrik Nielsen, Niels Pallisgaard, Mads Thomassen, Vibe Skov, Hans Carl Hasselbalch

**Affiliations:** ^1^ Department of Hematology Zealand University Hospital Roskilde Denmark; ^2^ Department of Hematology Rigshospitalet Copenhagen Denmark; ^3^ Department of Hematology Herlev University Hospital Copenhagen Denmark; ^4^ Institute for Inflammation Research Rigshospitalet Copenhagen Denmark; ^5^ Department of Clinical Genetics Odense University Hospital Odense Denmark; ^6^ Department of Hematology Odense University Hospital Odense Denmark; ^7^ Department of Hematology South‐West Jutlandic Hospital Esbjerg Denmark; ^8^ Department of Pathology Zealand University Hospital Roskilde Denmark

**Keywords:** combination therapy, interferon‐alpha, myeloproliferative neoplasms, polycythemia vera, primary myelofibrosis, ruxolitinib

## Abstract

Interferon‐α2 reduces elevated blood cell counts and splenomegaly in patients with myeloproliferative neoplasms (MPN) and may restore polyclonal hematopoiesis. Its use is limited by inflammation‐mediated toxicity, leading to treatment discontinuation in 10‐30% of patients. Ruxolitinib, a potent anti‐inflammatory agent, has demonstrated benefit in myelofibrosis (MF) and polycythemia vera (PV) patients. Combination therapy (CT) with these two agents may be more efficacious than monotherapy with either, potentially improving tolerability of interferon‐α2 as well. We report the preliminary results from a phase II study of CT with pegylated interferon‐α2 and ruxolitinib in 50 MPN patients (PV, n = 32; low‐/intermediate‐1‐risk MF, n = 18), the majority (n = 47) being resistant and/or intolerant to interferon‐α2 monotherapy. Objectives included remission (2013 revised criteria encompassing histologic, hematologic, and clinical responses), complete hematologic response (CHR), molecular response, and toxicity. Follow‐up was 12 months. Partial remission (PR) and sustained CHR were achieved in 9% and 44% of PV patients, respectively. In MF patients, complete or partial remission was achieved in 39%, and sustained CHR in 58%. The median *JAK2*V617F allele burden declined significantly in both groups. Hematologic toxicity was the most common adverse event and was managed by dose reduction. Thirty‐seven serious adverse events were recorded in 23 patients; the discontinuation rate was 20%. We conclude that CT with interferon‐α2 and ruxolitinib is efficacious in patients with low‐/intermediate‐1‐risk MF and, to a lesser extent, in patients with PV. These preliminary results encourage phase III studies as well as a study with CT in newly diagnosed MPN patients.

## 1 INTRODUCTION

The Philadelphia chromosome‐negative chronic myeloproliferative neoplasms (MPN) ‐ essential thrombocythemia (ET), polycythemia vera (PV) and primary myelofibrosis (PMF) ‐ are acquired hematopoietic stem cell neoplasms characterized by virtually mutually exclusive driver mutations in *Janus Kinase 2* (*JAK2*), *calreticulin* (*CALR*), and *myeloproliferative leukemia virus oncogene* (*MPL*). These mutations lead to constitutive activation of the *JAK*‐signal transducer and activator of transcription (STAT) signaling pathways promoting clonal myeloproliferation, production of inflammatory mediators, and progressive myelofibrosis.^1^ Chronic inflammation and a dysregulated immune system with impaired tumor surveillance are considered of importance in the pathogenesis and clonal evolution of MPN.^2^

The *JAK1‐2* inhibitor ruxolitinib is a potent anti‐inflammatory agent and has shown promising results in the treatment of patients with MF^3^, ^4^, ^5^ and PV^6^ in regard to reducing splenomegaly, constitutional symptoms, and inflammation‐mediated comorbidities. However, in the large majority of patients, ruxolitinib does not markedly reduce the *JAK2*V617F allele burden (%V617F).^5^, ^6^, ^7^

Interferon‐α2 (IFNα2) has potent antiproliferative and immunomodulatory effects and is devoid of suspicion of leukemogenicity unlike most available cytoreductive agents.^8^ Pegylated forms of IFNα2 (PEG‐IFNα2) allow administration once weekly and have proven efficacious in ET^9^, ^10^, ^11^, ^12^ and PV^9^, ^10^, ^11^, ^12^, ^13^, ^14^, ^15^. Patients in the early phase of MF, defined by low‐ or intermediate‐risk disease on the Dynamic International Prognostic Scoring System (DIPSS) scale,^16^ have also been shown to benefit from treatment with IFNα2.^9^, ^17^, ^18^ Of note, in a subset of patients, long‐term treatment with IFNα2 induces deep molecular remission sustained even years after cessation of therapy.^9^, ^10^, ^11^, ^13^, ^14^, ^15^ However, the use of IFNα2 is limited by toxicity with an average discontinuation rate of 10‐30%.^10^, ^12^, ^19^ Moreover, in a subset of MPN patients molecular responses are absent, which is in part attributed to additional non‐driver mutations and, possibly, concurrent inflammation and oxidative stress.^10^, ^20^

The hypothesized role of chronic inflammation in the progression of MPN and in attenuating the efficacy of IFNα2 implies that combination therapy (CT) with IFNα2 and ruxolitinib may be a rational strategy in patients with MPN.^2^, ^20^, ^21^ This approach may also improve tolerability of IFNα2 by allowing a lower dosage and reducing the inflammation‐mediated adverse effects. The proof‐of‐concept of this therapeutic strategy was published recently,^22^ and it has been described as one of the most promising in MPN.^23^

Herein, we report an interim analysis of the ongoing phase II trial, the COMBI study, evaluating safety and efficacy of CT with low‐dose PEG‐IFNα2 and ruxolitinib in 50 patients with PV or low‐/intermediate‐risk MF at 12 months of follow‐up.

## 2 PATIENTS AND METHODS

### 2.1 Study design

The COMBI study (#EudraCT2013‐003295‐12) is a prospective, open‐label, single‐arm phase II study conducted at three centers in Denmark from June 2014 and ongoing. Enrollment is completed, and a total of 51 patients have initiated study medication. Treatment duration will be maximum 24 months. The study was approved by the Danish Regional Science Ethics Committee and conducted in accordance with the principles of the Declaration of Helsinki. All patients provided written informed consent. Study medication was self‐administered and distributed in an out‐patient setting.

### 2.2 Inclusion and exclusion criteria

Inclusion criteria were age ≥18 years and a diagnosis of PV or MF of low‐ or intermediate‐1 or ‐2‐risk on the DIPSS scale,^16^ including PMF, post‐PV MF, and post‐ET MF according to, respectively, the 2008 WHO criteria^24^ and the International Working Group‐Myeloproliferative Neoplasms Research and Treatment (IWG‐MRT) criteria.^25^ Evidence of active disease was required, defined by a need for phlebotomy, white blood cell (WBC) count ≥10 × 10^9^/L, platelet count >400 × 10^9^/L, constitutional symptoms, pruritus, symptomatic splenomegaly, or previous thrombosis. A minor albeit not absolute criterion was intolerance and/or unresponsiveness to previous treatment with IFNα2.

Exclusion criteria were pregnancy, allergic hypersensitivity to any of the study medications, Eastern Cooperative Oncology Group (ECOG) performance status ≥3, other active malignancy within the past five years, impaired renal or hepatic function (serum creatinine >2 ×  the upper limit of the normal range (ULN), total serum bilirubin >1.5 ×  ULN, serum alanine transaminase >3 ×  ULN), chronic hepatitis with decompensated cirrhosis or treatment with immunosuppressive drugs (with the exception of corticosteroids) within the preceding six months, former psychiatric disease, severe neurologic disease, uncontrolled metabolic disease, severe cardiac disease (NYHA class 3‐4), leukopenia (WBC count <1.5 × 10^9^/L), or thrombocytopenia (platelet count <100 × 10^9^/L).

### 2.3 Study endpoints

The primary endpoint was complete (CR) or partial (PR) remission assessed at 12 months of follow‐up (the interim analysis) and at 24 months of follow‐up (the final analysis).

Secondary endpoints included toxicity, complete hematologic response (CHR), complete (CMR) or partial (PMR) molecular remission, and responses in individual hematologic parameters, splenomegaly, and patient‐reported outcomes (PRO).

Remission definitions in PV were in accordance with the European LeukemiaNet (ELN) and IWG‐MRT 2013 revised criteria.^26^ In MF, remission was defined by the IWG‐MRT and ELN 2013 revised criteria.^27^ These remission definitions encompass histologic, hematologic, and clinical responses (Appendix S1).

CHR was defined in accordance with the criteria for peripheral blood count remission in the 2013 revised response criteria^26^, ^27^ (Appendix S1).

Evaluation of toxicity followed the National Cancer Institute Common Terminology Criteria for Adverse Events version 4.^28^ Tolerability to therapy was inversely assessed by discontinuation rate.

The *JAK2*V617F allele burden was assessed by quantitative real‐time polymerase chain reaction performed on DNA purified from peripheral blood granulocytes with the recommended ELN/MPN&MPNr‐EuroNet *JAK2*V617F assay.^29^ Molecular remission definitions were in accordance with the ELN and IWG‐MRT 2013 criteria^26^ (Appendix S1).

PRO endpoints are described in Appendix S2.

### 2.4 Treatment

Initial therapy was PEG‐IFNα2a (Pegasys^®^; Genentech (Roche), South San Francisco, CA, USA) 45 μg or PEG‐IFNα2b (PegIntron^®^; Merck Sharp & Dohme, Hertfordshire, UK) 35 μg once weekly subcutaneously and ruxolitinib (Jakavi^®^; Novartis, Basel, Switzerland) 20 mg BID orally, the ruxolitinib dose being dependent on the platelet count. The doses and dose schedules were modified based on toxicity or lack of efficacy. If needed, patients were phlebotomized to reach the target hematocrit of <0.45 (males) or <0.42 (females). All patients received aspirin 75 mg daily unless contraindicated. Baseline investigations and initiation of CT were preceded by a wash‐out period of seven days from discontinuation of previous MPN‐directed therapy.

### 2.5 Evaluations

Follow‐up study visits were scheduled at baseline, 2 weeks, 1 month, 3 months, and every 3 months thereafter until 24 months = end of treatment. Investigations performed during follow‐up are described in Appendix S3.

### 2.6 Statistical analyses

The follow‐up time was 12 months. Efficacy was evaluated by use of a modified intention‐to‐treat (ITT) analysis. This method excluded patients who were deemed ineligible early after enrollment. For the calculation of median values, the per‐protocol study population was used, excluding patients who at a previous time point had discontinued study medication, and patients with a missing measurement.

Efficacy was primarily assessed using descriptive statistics. The Wilcoxon matched‐pairs signed rank test was used for comparing paired quantitative variables and Fisher's exact test for contingency analysis of response groups. *P* values <.05 denoted statistical significance. Analyses were performed using the GraphPad Prism 7.0 software (San Diego, CA, USA).

Sample size calculation: We set the expected proportion of patients achieving the primary endpoint with CT to 0.45 (estimated), the reference value to 0.27 (proportion of patients achieving the primary endpoint with IFNα2 monotherapy^30^), the power to 0.8 and the type I error rate to 0.05. Accordingly, our sample size should be 41 patients (One‐Sample Proportion Test, One‐Sample, One‐Sided).^31^

## 3 RESULTS

Of the 51 enrolled patients who started study medication, one patient with *CALR*‐mutated PMF died from transformation to acute myeloid leukemia (AML) after receiving the first dose of study medication and before reaching the 2 weeks study visit. This patient was included in the safety analyses but not in the efficacy analyses or patient demographics.

### 3.1 Patient characteristics

#### 3.1.1 Polycythemia vera

Thirty‐two patients (64%) had PV. Baseline demographics and disease characteristics of the patients are summarized in Table 1. All patients had received MPN‐directed therapies prior to enrollment, although one patient was not in active treatment at the time of enrollment. One patient was enrolled within six weeks of diagnosis due to a heavy disease burden with a frequent phlebotomy requirement despite cytoreductive therapy with hydroxyurea (HU).

**Table 1:** Baseline demographic and clinical characteristics

Parameter	Diagnosis	
	Polycythemia vera	Myelofibrosis
Number of patients, n (%)	32 (64)	18 (36)
		PMF 13 (72)
		Post‐PV MF 4 (22)
		Post‐ET MF 1 (6)
Risk category on the DIPSS scale, n (%)		
Low	n/a	7 (39)
Intermediate‐1	n/a	11 (61)
Intermediate‐2	n/a	0
Years of age, median (range)	57 (36‐78)	59 (39‐72)
Gender, male/female, n (%)	19/13 (59/41)	10/8 (56/44)
Years from diagnosis to inclusion, median (range)	7.0 (0.1‐24.3)	6.0 (0.1‐17.2)
*JAK2*V617F mutation positive, n (%)	32 (100)	12 (67)
*%JAK2*V617F at baseline, median (range)	47 (1.8‐97)	45 (0.1‐97)
Previously treated with IFNα2, n (%)	30 (94)	17 (94)
Intolerant to IFNα2 monotherapy	19 (59)	11 (61)
Refractory to IFNα2 monotherapy	7 (22)	4 (22)
A combination of the above	4 (13)	2 (11)
Latest MPN therapy prior to enrollment^a^, n (%)		
IFNα2	13 (41)	7 (39)
Hydroxyurea	9 (28)	2 (11)
Anagrelide	2 (6)	3 (17)
Ruxolitinib	0	1 (5)
Combination therapy (≥2 of the agents above) including IFNα2	7 (22)	3 (17)^b^
No medical treatment	1 (3)	2 (11)^c^
Hematologic parameters at baseline, median (range)		
Hematocrit, vol. fr.	0.43 (0.35‐0.48)	0.40 (0.31‐0.46)
WBC count, x10^9^/L	8.8 (3.3‐40.1)	7.8 (5.0‐15.7)
Platelet count, x10^9^/L	401 (69‐1010)	419 (143‐938)
Ultrasonographic spleen size (longest diameter in cm) at baseline, median (range)	13.8 (9.5‐30)^d^	14.0 (8‐29)
Splenomegaly at baseline, n (%)		
By ultrasonography^e^	19 (59)	11 (61)
By palpation	6 (19)	3 (17)
Size of splenomegaly at baseline, median (range)		
By ultrasonography, longest diameter in cm	14.5 (13.1‐30)	17.9 (13.5‐29)
By palpation, cm below the LCM	7.5 (2‐21)	18 (18)

DIPSS, Dynamic International Prognostic Scoring System; JAK, Janus kinase; IFN, interferon; MPN, myeloproliferative neoplasm; WBC, white blood cell; LCM, left costal margin; *n/a*, not applicable.

a During the preceding six months.

b Including one MF patient treated with combination therapy of anagrelide and hydroxyurea.

c Including one MF patient without medical treatment for one year (previously treated with IFNα2).

d One PV patient splenectomized. In another PV patient, no baseline imaging of spleen available (not performed). These two patients not included.

e Defined by longest diameter >13 cm.

#### 3.1.2 Myelofibrosis

Of the 50 patients included in the efficacy analyses, 18 (36%) had MF (Table 1). One patient with post‐PV MF was MPN treatment‐naïve and was enrolled shortly after the time of diagnosis due to inflammation‐mediated comorbidity (polymyalgia rheumatica) and, thus, a possible benefit of a CT with ruxolitinib. Six patients were non‐mutated in *JAK2* of which four had mutations in *CALR*, and two patients had neither mutations in *JAK2*,* CALR*, or *MPL* (triple‐negative MF).

### 3.2 Complete and partial remission

#### 3.2.1 Polycythemia vera

**Figure 1:**
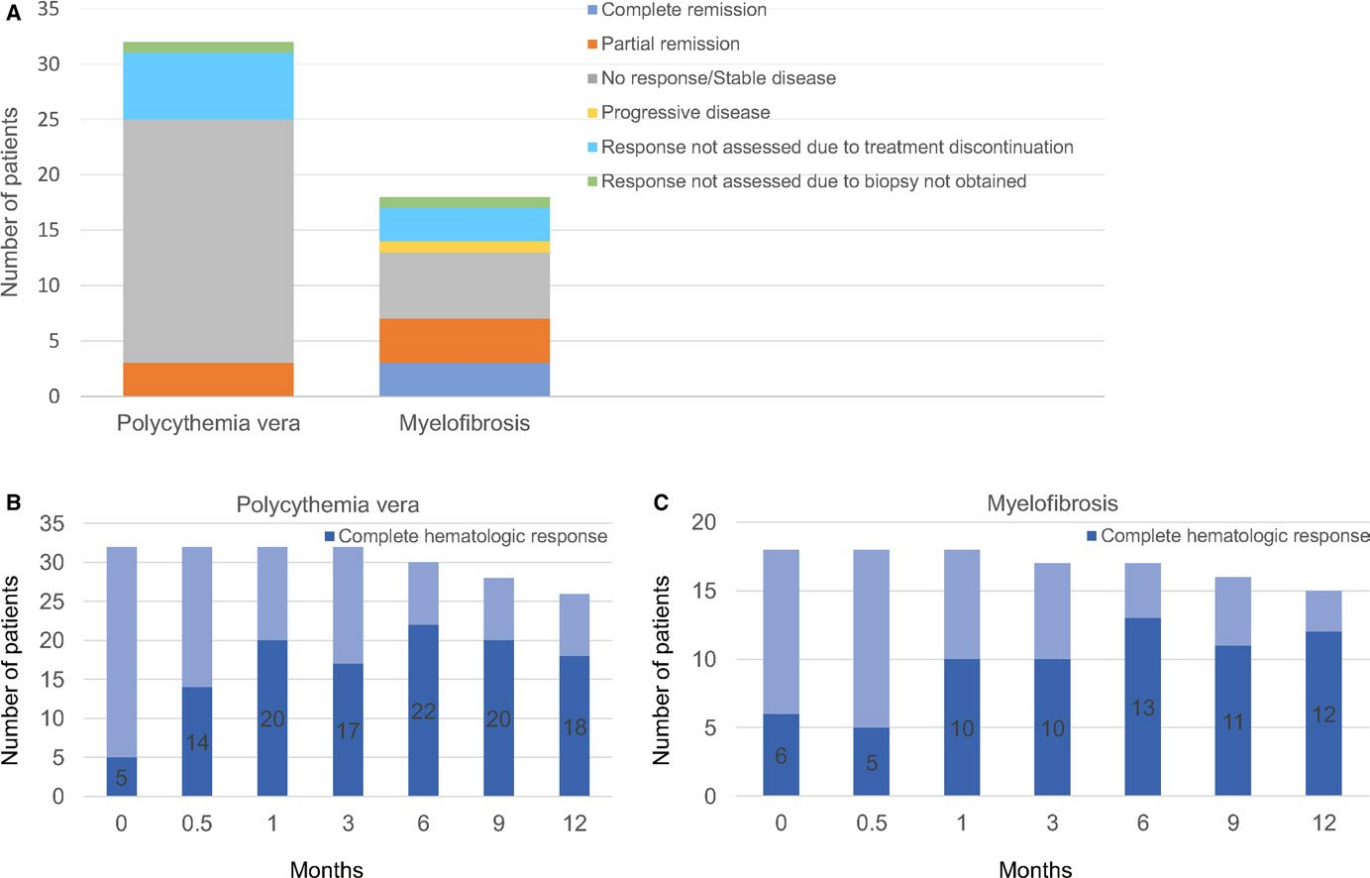
(A) The distribution of patients in response categories as defined by the ELN and IWG‐MRT 2013 revised response criteria (including histologic, hematologic, and clinical response) at 12 months of follow‐up. PV patients, n = 32; MF patients, n = 18. (B, C) The fraction of patients in complete hematologic response (defined in Appendix S1) at baseline (0 months), 2 weeks (0.5 months), 1 month, 3 months, 6 months, 9 months, and 12 months

CR was not achieved in any of the 32 patients. PR was achieved in three patients (9%; Appendix S4a); 22 patients (69%) had no response (Figure 1A). Six patients discontinued study medication before 12 months of follow‐up and were not evaluated for remission in the absence of a control bone marrow biopsy. In one patient, an assessment of remission was not made since a control bone marrow biopsy was not performed (investigator's decision out of consideration for the patient). No patients had progressive disease. However, five of the non‐responding patients had marrow morphology consistent with post‐PV MF at 12 months of follow‐up (in two patients unchanged from baseline; in three patients representing progression from a marrow morphology consistent with PV at baseline), but did not fulfill the additional criteria for a diagnosis of post‐PV MF either at baseline or at 12 months of follow‐up (Appendix S4a). Three of the non‐responding patients fulfilled the criteria for histologic remission, but did not fulfill one or more of the other remission criteria.

Two of the three patients who achieved PR had non‐palpable spleens at baseline. In one patient achieving PR, baseline palpable splenomegaly was present at 5 cm, below the left costal margin (LCM).

#### 3.2.2 Myelofibrosis

CR was achieved in 3 of the 18 patients (17%; PMF, n = 1; post‐PV MF, n = 1; post‐ET MF, n = 1), and PR was achieved in four patients (22%; PMF, n = 3; post‐PV MF, n = 1; Figure 1A; Appendix S4b). Six patients (33%) had stable disease, and one patient (6%) with PMF had progressive disease (a significant increase in spleen volume from baseline; Appendix S4b). Three PMF patients, of whom two harbored *CALR* mutations, discontinued study medication before 12 months of follow‐up and were not evaluated for remission. In one patient, also *CALR*‐mutated, an assessment of remission was not made, since a control marrow biopsy was not performed due to risk of bleeding under antiplatelet therapy. The remaining patient of the four *CALR*‐mutated patients had stable disease. The two triple‐negative patients achieved CR and maintained stable disease, respectively.

All seven patients who achieved remission had non‐palpable spleens at baseline.

### 3.3 Complete hematologic response

#### 3.3.1 Polycythemia vera

Five patients were in CHR at baseline (Figure 1B). Counting only patients not in CHR at baseline, CHR was achieved at some point in 22 out of 27 patients (81%) and sustained ≥3 months including at 12 months of follow‐up in 12 patients (44%) with a median time to response of 1 month (range, 0.5‐6 months).

All five patients in CHR at baseline had disease‐related symptoms at baseline and, thus, evidence of active disease.

Responses in individual hematologic parameters, splenomegaly, and PROs are presented in Appendix S5a.

#### 3.3.2 Myelofibrosis

Six patients were in CHR at baseline (Figure 1C). Counting only patients not in CHR at baseline, CHR was achieved at some point in 11 out of 12 patients (92%) and sustained ≥3 months including at 12 months of follow‐up in 7 patients (58%; PMF, n = 4; post‐PV MF, n = 2; post‐ET MF, n = 1) with a median time to response of 3 months (range, 0.5‐9 months).

All six patients in CHR at baseline had constitutional symptoms at baseline.

Response in PROs is presented in Appendix S5b.

### 3.4 Molecular response

#### 3.4.1 Polycythemia vera

**Figure 2:**
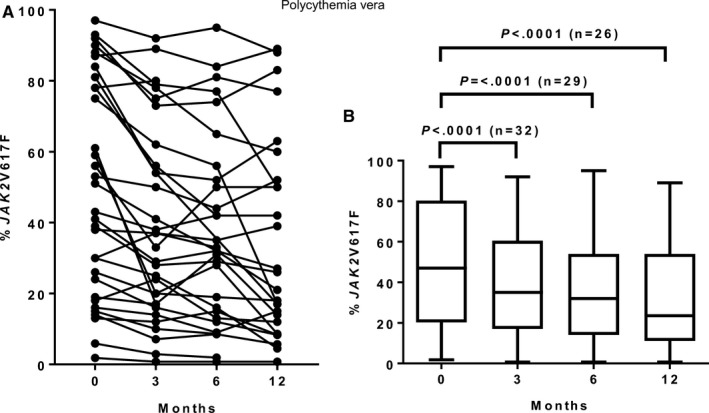
(A) Dynamics of the *JAK2*V617F allele burden (% *JAK2*V617F) in individual PV patients. (B) Median % *JAK2*V617F including range and 25 and 75 percentiles at baseline (0 months), 3 months, 6 months, and 12 months. One missing value in one patient at 6 months

A decline in %V617F was observed in 28 patients (88%) during follow‐up, with no evidence of a plateau in half of these patients (Figure 2A; Appendix S6a). The median %V617F decreased significantly from 47% (range, 1.8‐97%) at baseline to 23.5% (range, 0.73‐89%) at 12 months (*P *<* *.0001; Figure 2B). No patients achieved CMR; PMR was achieved in six patients (19%) by 3 months (n = 2), 6 months (n = 1), and 12 months (n = 3), respectively.

The three patients in PR at 12 months were among the six patients achieving PMR (50% PR in molecular responders versus 0% PR in molecular non‐responders; *P *=* *.004). The median baseline %V617F was not significantly different between the molecular responders (60%, range, 24‐84%) and the molecular non‐responders with %V617F ≥20 (n = 18; 67%, range, 26‐97%; *P *=* *.387).

#### 3.4.2 Myelofibrosis

**Figure 3:**
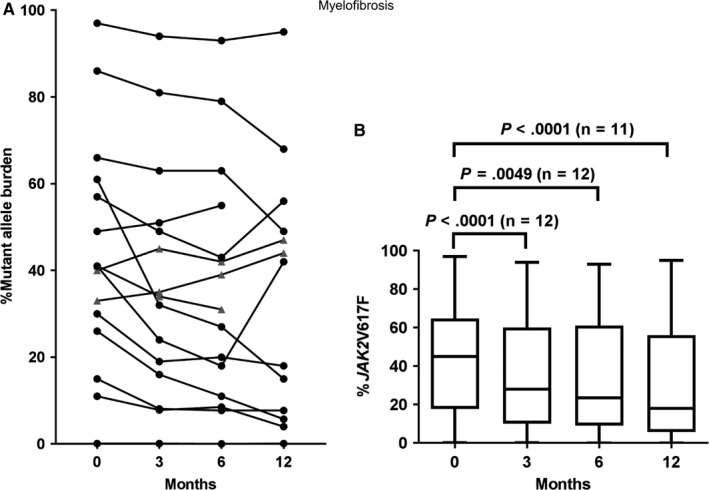
(A) Dynamics of the mutant allele burden in individual MF patients. (*black dot*) depicting mutant allele burden in *JAK2*‐mutated patients; (*grey triangle*) depicting mutant allele burden in *CALR*‐mutated patients, excluding one patient with discontinuation of study therapy after 1 month of follow‐up. (B) Median *JAK2*V617F allele burden (% *JAK2*V617F) including range and 25 and 75 percentiles at baseline (0 months), 3 months, 6 months, and 12 months

In 10 of the 12 *JAK2*‐mutated patients (83%), a decline in %V617F was observed during follow‐up with no evidence of a plateau in half of these patients (Figure 3A; Appendix S6b). The median %V617F decreased significantly from 45% (range, 0.1‐97%) at baseline to 18% (range, 0.08‐95%) at 12 months (*P *<* *.0001; Figure 3B). No patients achieved CMR, although one PMF patient with a baseline %V617F of 0.1% had no detectable mutant clone at 6 months but became detectable again at 12 months at 0.08%. PMR was achieved in two patients (17%) with PMF and post‐PV MF, respectively, by 6 months with a further decline in %V617F at 12 months. One additional patient achieved non‐sustained PMR at 6 months; at 12 months of follow‐up, %V617F had increased to baseline level following a pause of PEG‐IFNα2 for 3 months due to headache.

Both patients achieving sustained PMR were in PR at 12 months (100% PR in molecular responders versus 40% CR/PR in molecular non‐responders; *P *=* *.125).

The dynamics of the mutant allele burden in the *CALR*‐mutated patients are depicted in Figure 3A and listed in Appendix S6b.

### 3.5 Toxicity

Fifty‐one patients (PV, n = 32; MF, n = 19) were included in the safety analyses. Hematologic toxicities were the most common adverse events (AE) and were managed by dose reduction or pausing of study medication. Anemia (PV, 56%; MF, 74%), leukopenia (PV, 50%; MF, 26%), and neutropenia (PV, 0%; MF, 16%) were in all patients of grade 1‐2 while thrombocytopenia (PV, 28%; MF, 32%) of grade ≥3 was reported in one PV patient (platelet count 13 × 10^9^/L).

The most common non‐hematologic AEs, regardless of causality, were arthralgia and/or myalgia (n = 40, in 24 patients), symptoms related to PEG‐IFNα2 injection (flu‐like symptoms, injection site reactions; n = 36, in 24 patients), and gastrointestinal symptoms (nausea, abdominal pain, dyspepsia, diarrhea; n = 31, in 27 patients; Appendix S7).

Thirty‐seven serious AEs (SAE) were recorded in 23 patients (45%; Table 2). All SAEs, except for conversion to AML resulting in death, were defined so by consequent hospital admission. Four SAEs; angina pectoris, herpes zoster (n = 2), and anemia/thrombocytopenia, were considered causally related to study therapy, with the angina pectoris patient and one patient with herpes zoster leading to treatment discontinuation. One thromboembolic event (ischemic stroke) was recorded in a PMF patient during the follow‐up period.

**Table 2:** Serious adverse events

Serious adverse event	Polycythemia vera, n	Myelofibrosis, n
Fever	1 (+vomiting)	5^b^ (+cystitis in 1 case)
Pneumonia	4	2
Bacterial infection, not otherwise specified	1	2^a^ (+anemia)
Melena		3^b^
Extremity pain	1	1
Angina pectoris	1^b^,1	
Arterial hypertension	2^a^	
Herpes zoster	2^b^	
Conversion to AML		1 (fatal outcome)
Ischemic stroke		1
Lipotymia	1	
Diverticulitis with sepsis		1
Dehydratio	1	
Phlebitis	1	
Peripheral facial palsy	1	
Anemia, thrombocytopenia		1^b^
Dyspnea	1	
Influenza		1
Operative removal of bladder tumor	1	
Total	19	18
Number of patients with ≥1 serious adverse event(s) (%)	15 (47)	8 (42)

AML, acute myeloid leukemia.

PV patients, n = 32; MF patients, n = 19.

In 2 patients.

a In 1 patient.

b Considered causally related to study therapy.

**Figure 4:**
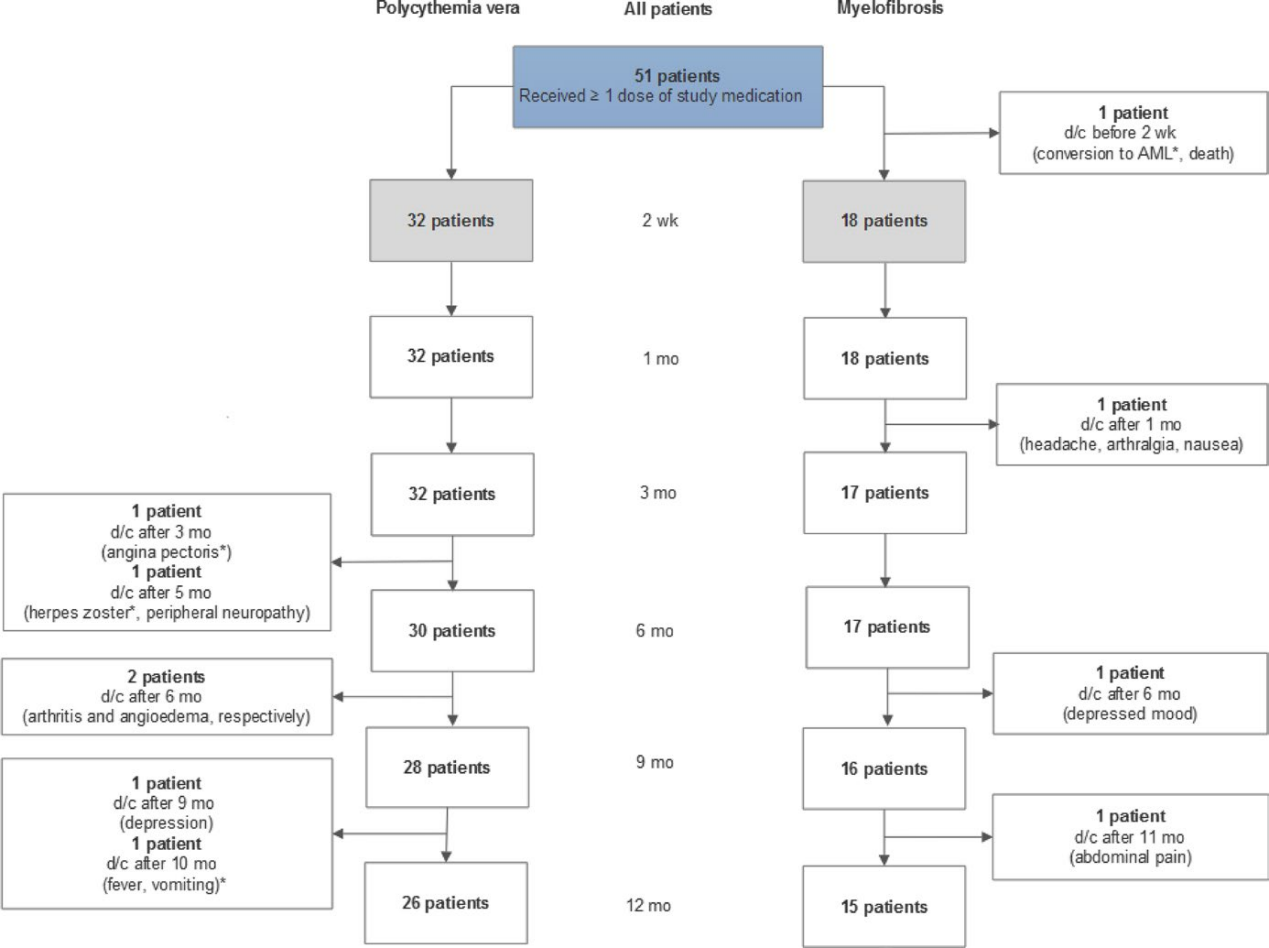
Patient distribution and representation of patient discontinuations of study medication. () the (serious) adverse event(s) leading to discontinuation of study therapy, *serious adverse event. d/c, discontinuation of study medication

Twenty‐six PV patients (81%) and 15 MF patients (79%) remained on study medication at 12 months of follow‐up (Figure 4).

Dose modifications and median doses of study medication are presented in Appendix S8a,b.

## 4 DISCUSSION

In this first study of CT with PEG‐IFNα2 and ruxolitinib in patients with PV (n = 32) or low‐/intermediate‐risk MF (n = 18) ‐ mainly patients being intolerant and/or refractory to IFNα2 monotherapy (94%) ‐ remission (ELN and IWG‐MRT 2013 revised response criteria including histologic, hematologic, and clinical responses^26^, ^27^) was achieved at 12 months of follow‐up in a subset of PV patients (9%, PR) and a large fraction of MF patients (39%, including CR in 17%) in concert with a significant decline in the median %V617F. A high number of SAEs (n = 37, in 23 patients), but no unexpected toxicities, were reported, and the discontinuation rate was 20%. One thromboembolic event was reported, and one case of progressive disease, both in PMF patients.

An advantage of this pioneering study was the multiparametric evaluation of treatment efficacy in both PV and MF patients. Furthermore, international consensus‐based remission definitions were applied, with remission criteria not only encompassing improvement in or normalization of peripheral blood counts and spleen size, as used in multiple prior studies,^9^, ^10^, ^13^ but also of marrow morphology and disease‐related symptoms. The 2013 revised response criteria were published after the preparation of the study protocol; however, for the evaluation of efficacy in this interim analysis, we chose to adopt these. The revised response criteria correlate better with measures of benefit for the patients than the 2009 criteria originally intended as the primary endpoint of this study. However, it was necessary to modify the 2013 criteria by not including an assessment of palpable hepatomegaly, and in MF by confirming changes in spleen size by ultrasonography instead of MRI/CT since these investigations were not performed.

Our study has other limitations to consider. Firstly, the cohort size of MF patients, in particular, is relatively small, and the follow‐up period is short, which hinders the detection of rare or long‐term toxicities, as well as the evaluation of depth and long‐term sustainability of responses. Secondly, the initial dosage of ruxolitinib of 20 mg BID differs from the recommended 10 mg BID for PV patients, because the ruxolitinib dose‐finding study^32^ had not been published by the time the protocol for this study was prepared. However, in the majority of patients, doses were decreased according to peripheral blood counts. Thirdly, we applied a modified ITT analysis instead of a true ITT. Separating the modified ITT analysis from a true ITT analysis is the exclusion of screen failure patients deemed ineligible due to failure to meet the in‐/exclusion criteria (patients who never received study medication), and the exclusion of the one MF patient who died from transformation to AML within two weeks of study start (erroneous implementation of eligibility criteria). This patient was, however, included in the safety analyses. In terms of presenting clinically meaningful analyses of the efficacy and safety of the study therapy giving maximum information to clinicians, we consider the use of the modified ITT analysis to be a rational approach without risking bias.^33^

Fourthly, based on the median values for baseline peripheral blood counts (Table 1), one could argue that the patient population, seemingly having a low burden of disease, was not representative. However, these median baseline values were influenced by a high number of prior phlebotomies and a relatively short preceding wash‐out period of seven days. Eleven patients in total (22%; PV, n = 5; MF, n = 6) were in CHR (based solely on peripheral blood counts) at baseline, all of them having disease‐related/constitutional symptoms at baseline.

These limitations notwithstanding, our preliminary results are remarkable since few prior studies in MPN have shown remission rates (by the revised definitions) of this amplitude at one year of follow‐up.

In a prospective study of IFNα2 monotherapy in patients with PMF, post‐PV MF, or post‐ET MF of low‐ or intermediate‐1‐risk disease, CR and PR (2013 IWG‐MRT and ELN criteria) were achieved in 7% (2/30) and 30% (9/30) of patients, respectively, with a median therapy duration in the cohort of 5.6 years.^18^

In the context of the encouraging results of this CT, it is relevant to consider, if similar results might be obtained with ruxolitinib alone. Monotherapy with ruxolitinib in phlebotomy‐dependent PV patients with splenomegaly who were resistant or intolerant to HU was in a phase III study associated with CHR in 24% of patients after eight months.^6^ In our study, sustained CHR was achieved in 44% of PV patients and 58% of MF patients, who were not in CHR at baseline, by a median follow‐up time of 1 month (range, 0.5‐6 months) and 3 months (range, 0.5‐9 months), respectively. Furthermore, ruxolitinib has no or minor impact on the malignant clone but primarily on the inflammatory process associated with MPNs. Thus, we suggest that CT targeting both the malignant clone (IFNα2) and the inflammatory state (ruxolitinib) may be superior to monotherapy with ruxolitinib as described in most recent reviews.^2^, ^21^, ^23^

HU is the most widely used cytoreductive agent internationally and is efficient in reducing elevated peripheral blood counts and preventing thrombosis. However, studies of the effect of HU on the *JAK2*V617F allele burden have shown divergent results.^34^ In this regard, the direct prognostic relevance of a reduction in the *JAK2*V617F allele burden is not fully elucidated, however, a higher allele burden seems to be associated with a more advanced disease stage and more symptoms, a higher risk of disease evolution, and an increased risk of thrombosis.^35^, ^36^ Thus, the reduction in allele burden is considered a clinically relevant endpoint and a valid surrogate marker for treatment efficacy.

We found that remission rates were higher among patients in PMR than among patients not in PMR (significant only for PV patients), and higher remission rates were also observed among patients with >50% reduction in total symptom score within the first 2 weeks of therapy than among patients without (non‐significant; Appendix S5a,b). In regard to the former, results from other studies are divergent.^37^, ^38^ In addition, remission was achieved only in patients with baseline palpable splenomegaly ≤5 cm, below the LCM, supporting the findings of other studies.^39^

A finding that must be stressed is the high number of SAEs. However, a large proportion of hospitalizations eliciting SAEs were effectuated as a precaution in the context of an experimental CT rather than reflecting severely affected patients. Accordingly, many hospitalizations were terminated within 24 hours. Some of these hospitalizations might have been explained by the initial dosage of ruxolitinib of 20 mg BID which we also underscore as a limitation of our study. In future studies of this combination therapy, the recommended 10 mg BID for PV patients should be used initially,^32^ thereby hopefully reducing the incidence of SAE's.

Moreover, 94% of patients were intolerant and/or refractory to IFNα2 monotherapy, yet tolerability of CT was comparable to the one reported with low‐dose PEG‐IFNα2 monotherapy in IFNα2‐naïve patients.^12^

It is reasonable to question a possible synergistic effect of combining IFNα2 and ruxolitinib, when ruxolitinib by inhibiting JAK1 and JAK2 and, thereby, the JAK‐STAT‐signaling pathway, might actually prohibit IFNα2 signaling which is mediated through primarily JAK1. However, ruxolitinib has a short half‐life of approximately 3 hours as opposed to PEG‐IFNα2 with a prolonged half‐life of several days. This leaves a time window of several hours daily, in which IFN signaling is possible.^21^ However, a JAK2‐selective inhibitor might synergize more with IFNα2 and should be explored in future studies.

In conclusion, the preliminary results from this study suggest that CT with low‐dose PEG‐IFNα2 and ruxolitinib is feasible and efficacious in patients with low‐/intermediate‐risk MF and, to a lesser extent, in patients with PV, including in patients who were unresponsive or intolerant to IFNα2 monotherapy. These results warrant phase III studies to assess the safety and efficacy of CT compared to current standard treatment modalities as well as a study with CT in patients with newly diagnosed PV or low‐/intermediate‐risk MF. In these studies, lower initial dosages of ruxolitinib (10 mg BID for PV patients) and PEG‐IFNα2a (Pegasys^®^ 45 ug once weekly or every second week subcutaneously) should preferentially be used in order to reduce the toxicity of this novel combination therapy.

## CONFLICT OF INTEREST DISCLOSURE

Stine Ulrik Mikkelsen and Niels Pallisgaard have received a travel grant from Novartis Oncology. Hans Carl Hasselbalch, Claus Henrik Nielsen, Niels Pallisgaard, and Mads Emil Bjørn have received research funding from Novartis Oncology. Niels Pallisgaard is in the Novartis Speakers Bureau. Ole Weis Bjerrum and Daniel El Fassi have conducted educational activities for Novartis. All other authors have no relationships to disclose.

## AUTHOR CONTRIBUTIONS

Stine Ulrik Mikkelsen: Writing ‐ original draft, writing ‐ review and editing, data curation, investigation, project administration, and visualization. Lasse Kjær: Data curation, formal analysis, investigation, resources, software, and visualization. Mads Emil Bjørn: Conceptualization, investigation, methodology, and writing ‐ review and editing. Trine Alma Knudsen: Investigation. Anders Lindholm Sørensen: Investigation, and project administration. Christen Lykkegaard Andersen: Conceptualization, methodology, and resources. Ole Weis Bjerrum: Conceptualization, resources, and writing ‐ review and editing. Nana Brochmann: Investigation, methodology, and resources. Daniel El Fassi: Conceptualization, resources, and writing ‐ review and editing. Torben A Kruse: Conceptualization, and resources. Thomas Stauffer Larsen: Conceptualization, investigation, and writing ‐ review and editing. Hans Torben Mourits‐Andersen: Investigation. Claus Henrik Nielsen: Conceptualization, investigation, and resources. Niels Pallisgaard: Conceptualization, methodology, and resources. Mads Thomassen: Resources. Vibe Skov: Investigation, resources, and writing ‐ review and editing. Hans Carl Hasselbalch: Conceptualization, funding acquisition, investigation, methodology, project administration, supervision, validation, and writing ‐ review and editing.
