# Supplementary material for: Safety and efficacy of combination therapy of interferon‐α2 and ruxolitinib in polycythemia vera and myelofibrosis
Source: Cancer Med. 2018 Jun 22;7(8):3571–81. doi: 10.1002/cam4.1619 (PMC6089176; doi:10.1002/cam4.1619)
Supplement: Supplementary file 1 [file CAM4-7-3571-s001.pdf]

Supporting Information, Appendix S1

Polycythemia vera, remission criteria[1]

CR was defined by bone marrow histologic remission (presence of age-adjusted normocellularity; disappearance of trilinear hyperplasia; absence of > grade 1 reticulin fibrosis), peripheral blood count remission (hematocrit < 0.45 without phlebotomy; WBC count < 10 x 10^9/l; platelet count ≤ 400 x 10^9/l), and response in disease-related signs and symptoms (absence of palpable (hepato)splenomegaly; ≥ 10-point decrease in MPN Symptom Assessment Form (MPN-SAF) Total Symptom Score (TSS)). **PR** was defined by fulfillment of the above listed criteria except for bone marrow histologic remission. Achievement of remission required durability of responses for ≥ 12 weeks (except in bone marrow histology), and absence of any hemorrhagic or thrombotic event, and absence of progressive disease. **Progressive disease** was defined by transformation into post-PV MF, myelodysplastic syndrome, or acute leukemia. Any response not meeting the criteria for the above listed response categories was classified as **no response**.

For the purpose of this analysis, the criteria were modified by not including an assessment of palpable hepatomegaly.

Myelofibrosis, remission criteria[2]

CR was defined by response in bone marrow histology (age-adjusted normocellularity; < 5% blasts; ≤ grade 1 fibrosis), in peripheral blood counts (hemoglobin ≥ 100 g/l and < UNL; neutrophil count ≥ 1 x 10^9/l and < UNL; platelet count ≥ 100 x 10^9/l and < UNL; < 2% immature myeloid cells), and in disease-related signs and symptoms (resolution of symptoms; absence of palpable (hepato)splenomegaly; no evidence of extramedullary hematopoiesis). **PR** was defined by response in peripheral blood counts and in disease-related signs and symptoms, or by response in bone marrow histology and in disease-related signs and symptoms and with hemoglobin 85-99 g/l, neutrophil count 1-7 x 10^9/l, platelet count 50-99 x 10^9/l, and < 2% immature myeloid cells. **Clinical improvement** was defined by response in anemia, in splenomegaly, or in disease-related symptoms without increase in the severity of anemia, thrombocytopenia, or neutropenia, and without progressive disease. **Progressive disease** was defined by leukemic transformation, or a new

splenomegaly confirmed by ultrasonography showing ≥ 25% increase in spleen volume from baseline. Any response not meeting the criteria for the above listed response categories was classified as **stable disease**.

For the purpose of this analysis, the criteria were modified by not including an assessment of palpable hepatomegaly, and by confirming changes in spleen size with ultrasonography instead of MRI/CT. Furthermore, baseline and posttreatment bone marrow slides were not interpreted at one sitting by a central review process as recommended/required in either PV or MF patients. Pathologists employed at the three sites participating in the study evaluated bone marrow morphology and histologic remission locally comparing baseline and posttreatment slides.

Polycythemia vera and myelofibrosis, molecular remission criteria[1]

CMR was defined as eradication/undetectability of a preexisting abnormality, and **PMR** was defined as ≥ 50% decrease from baseline in mutant allele burden (applicable only to patients with ≥ 20% mutant allele burden at baseline). Any response not meeting these criteria was classified as **no response**.

1. Barosi G, Mesa R, Finazzi G, et al. Revised response criteria for polycythemia vera and essential thrombocythemia: an ELN and IWG-MRT consensus project. *Blood*. 2013;121:4778-4781.

2. Tefferi A, Cervantes F, Mesa R, et al. Revised response criteria for myelofibrosis: International Working Group-Myeloproliferative Neoplasms Research and Treatment (IWG-MRT) and European LeukemiaNet (ELN) consensus report. *Blood*. 2013;122:1395-1398.

Supporting Information, Appendix S2

Patient-reported outcome endpoints

PROs, including individual symptoms, symptom burden, and quality of life, were assessed with the use of the validated MPN-SAF[3] and the co-administered Brief Fatigue Inventory (BFI)[4] translated into Danish in accordance with the principles of good practice.[5] According to patient preference, the questionnaires were filled out on paper or online in a secure software system.[6] The MPN-SAF includes 17 disease-related symptoms to be scored from 0 (absent) to 10 (worst imaginable) during the past week on a linear self-assessment scale, and a TSS (range 0-100) is calculated as the sum of the scores of the 10 most pertinent symptoms (problems with concentration, early satiety, inactivity, night sweats, itching, bone pain, abdominal discomfort, weight loss, fever, and "worst fatigue" from the BFI).[7] Treatment efficacy in TSS is included in this interim report in Appendix S5a,b.

3. Scherber R, Dueck AC, Johansson P, et al. The Myeloproliferative Neoplasm Symptom Assessment Form (MPN-SAF): International Prospective Validation and Reliability Trial in 402 patients. *Blood*. 2011;118:401-408.

4. Mendoza TR, Wang XS, Cleeland CS, et al. The rapid assessment of fatigue severity in cancer patients. *Cancer*. 1999;85:1186-1196.

5. Wild D, Grove A, Martin M, et al. Principles of Good Practice for the Translation and Cultural Adaptation Process for Patient-Reported Outcomes (PRO) Measures: Report of the ISPOR Task Force for Translation and Cultural Adaptation. *Value Health*. 2005;8:94-104.

6. Brochmann N, Zwisler A-D, Kjerholt M, Flachs EM, Hasselbalch HC, Andersen CL. A new internet-based tool for reporting and analysing patient-reported outcomes and the feasibility of repeated data collection from patients with myeloproliferative neoplasms. *Qual Life Res*. 2016;25:835-846.

7. Emanuel RM, Dueck AC, Geyer HL, et al. Myeloproliferative Neoplasm (MPN) Symptom Assessment Form Total Symptom Score: Prospective International Assessment of an Abbreviated Symptom Burden Scoring System Among Patients With MPNs. *J Clin Oncol*. 2012;30:4098-4103.

Supporting Information, Appendix S3

Evaluations

At each study visit, patient history was obtained, and physical examination including clinical assessment of splenomegaly was performed as well as blood tests (complete blood count; differential count; serum chemistries). The number of phlebotomies since last visit was recorded, at baseline the applicable time period being the preceding three months. The PRO questionnaires were completed within a week of each study visit. At baseline, 3, 6, 12, and 24 months/end of treatment, an ultrasonographic assessment of spleen size was performed, and the mutant allele burden was analyzed. Baseline studies further included bone marrow aspiration and biopsy with cytogenetics, which was repeated at 12 months and 24 months/end of treatment (cytogenetics only if abnormality had been detected). Cytogenetic response was not evaluated in this interim report.

Supporting Information, Appendix S4a

Baseline characteristics of PV patients achieving partial remission at 12 months of follow-up.

Patient number	Baseline						
	Years of age	Need for phlebotomy	WBC, x 10^9/l	Platelet count, x 10^9/l	Palpable splenomegaly, (cm below LCM)	Total symptom score	%V617F
10	34	No	14.7	585	No	71	59
29	46	No	17.3	413	Yes (5)	28	75
30	78	Yes*	13.4	254	No	1	84

*Phlebotomy performed on average every other week or more in the three months preceding baseline

Characteristics of PV patients with bone marrow morphology consistent with post-PV MF at 12 months of follow-up.

Patient number	Baseline							
	Years of age	Need for phlebotomy	WBC, x 10^9/l	Platelet count, x 10^9/l	Palpable splenomegaly, (cm below LCM)	Total symptom score	%V617F	Bone marrow fibrosis grade (0-3 scale)[8]
4*	68	No	18.3	216	Yes (21)	30	88	3
25*	75	Yes	10.8	339	Yes (10)	40	90	3
18	76	No	40.1	69	No	27	81	0
20	36	No	4.8	594	No	44	14	0-1
21	75	No	5.7	269	No	21	93	1
	12 months of follow-up							
	Need for phlebotomy/Anemia		WBC, x 10^9/l	Platelet count, x 10^9/l	Palpable splenomegaly, (cm below LCM)	Total symptom score	%V617F	Bone marrow fibrosis grade (0-3 scale)
4*	No/Yes		5.1	96	Yes (9)	18	60	3
25*	No/Yes		6.4	206	Yes (8)	15	77	3
18	No/Yes		15.9	23	No	16	63	2-3
20	No/No		4.4	384	No	50	5.7	2
21	No/Yes		5.0	268	No	9	50	2

Abbreviations (both tables): WBC, white blood cells; LCM, left costal margin

*Patients with bone marrow morphology consistent with post-PV MF at baseline without meeting the other criteria for post-PV MF

Supporting Information, Appendix S4b

Baseline characteristics of MF patients achieving complete or partial remission at 12 months of follow-up.

Patient number	Baseline									
	Diagnosis	Risk category, DIPSS scale	Years of age	Hemoglobin, g/l	WBC, x 10^9/l	Platelet count, x 10^9/l	Palpable splenomegaly	Total symptom score	%V617F	Bone marrow fibrosis grade (0-3 scale)
**Complete remission**										
37*	Post-PV MF	Intermediate-1	72	137	15.2	533	No	31	66	2
44	Post-ET MF	Low	50	134	11.3	421	No	8	30	1
49	PMF	Intermediate-1	50	139	7.5	376	No	36	*Triple-negative*	1-2
**Partial remission**										
36	PMF	Intermediate-1	56	106	8.2	548	No	7	0.1	3
39	PMF	Low	60	123	8.0	605	No	3	11	2
40	Post-PV MF	Intermediate-1	70	119	5.3	143	No	*Missing value (constitutional symptoms present)*	61	3
43	PMF	Low	62	127	5.5	459	No	8	26	2

*Within the first two weeks of study therapy, total symptom score declined to 11 (65% reduction) and the patient developed pancytopenia (hemoglobin 119 g/l, WBC 3.7 x 10^9/l, platelet count 79 x 10^9/l) necessitating pausing of study medication for 3 weeks

Characteristics of the MF patient with progressive disease at 12 months of follow-up. Description of the clinical course.

Patient number	Baseline									
	Diagnosis	Risk category, DIPSS scale	Years of age	Hemoglobin, g/l	WBC, x 10^9/l	Platelet count, x 10^9/l	Palpable splenomegaly, (cm below LCM)	Total symptom score	%V617F	Bone marrow fibrosis grade (0-3 scale)
41	PMF	Low	58	134	8.5	383	No	25	86	3
	12 months of follow-up									
		Intermediate-2		111*	6.8	217	Yes (19)	41	68	2-3

Abbreviations (both tables): DIPSS, Dynamic International Prognostic Scoring System[9]; WBC, white blood cells; PV, polycythemia vera; MF, myelofibrosis; ET, essential thrombocythemia; PMF, primary myelofibrosis; LCM, left costal margin

*Transfusion-dependent

Initially, the patient responded well to combination therapy achieving complete hematologic response at 1 month of follow-up. After 7 months of follow-up, over the course of a couple of

weeks, the patient developed pancytopenia including transfusion-dependent anemia, and an increase in spleen size from non-palpable to palpable 15 cm, below the LCM, between study visits 6 and 9 months. In addition, constitutional symptoms intensified, and the patient had a weight loss of 12 kg over two months. PEG-IFNα2a was paused for two months followed by dose reduction to 45 μg every other week, and the ruxolitinib dose was titrated down to 5 mg BID at 7 months of follow-up. At 8 months of follow-up, peripheral blood counts improved, although the patient remained anemic and transfusion-dependent. The patient was admitted to hospital three times between 7 and 8 months of follow-up with fever, failure to thrive, and an increase in C-reactive protein (CRP). Extensive diagnostic work-up, including PET/CT scan, did not show any infectious focus or pathology other than an increased metabolic activity in the bone marrow and an enlarged spleen. At 12 months of follow-up, further progression had occurred; the patient required 19 bags of packed red blood cells over the preceding 3 months, and splenomegaly palpable at 19 cm, below the LCM, was confirmed by ultrasonography with the longest diameter at 27 cm (19.5 cm at baseline). An interjected bone marrow biopsy at 7.5 months of follow-up showed a massively increased cellularity dominated by megakaryocytes with considerable atypia, decreased erythropoiesis, and reticulin fibrosis grade 2 (no collagen fibrosis). The myeloblast percentage had increased to 5-8% from 1-2% at baseline. It was concluded that disease progression towards AML was the cause of the patient's symptoms, and the patient was referred to a non-myeloablative allogeneic bone marrow transplantation. At 12 months of follow-up, the patient was still on combination therapy waiting for the bone marrow transplantation.

8. Thiele J, Kvasnicka HM, Facchetti F, Franco V, van der Walt J, Orazi A. European consensus on grading bone marrow fibrosis and assessment of cellularity. *Haematologica*. 2005;90:1128-1132.

9. Passamonti F, Cervantes F, Vannucchi AM, et al. A dynamic prognostic model to predict survival in primary myelofibrosis: a study by the IWG-MRT (International Working Group for Myeloproliferative Neoplasms Research and Treatment). *Blood*. 2010;115:1703-1708.

Supporting Information, Appendix S5a

I. Treatment efficacy in individual hematologic and clinical parameters in PV

Thirteen patients (41%) had an elevated hematocrit (≥ 0.45 in males or ≥ 0.42 in females) at baseline. The target hematocrit without phlebotomy was achieved in all 13 patients by a median follow-up time of 1 month (range, 2 weeks-6 months) and was sustained ≥ 3 months including at 12 months of follow-up in nine patients. Five phlebotomies were performed in three patients during follow-up compared to 27 phlebotomies in 14 patients in the three months prior to initiation of study medication. WBC count normalized in 9 of 11 patients, who at baseline had WBC count ≥ 10 x 10^9/l (median baseline WBC count 14.8 x 10^9/l, range 10.3-40.1 x 10^9/l, in the 11 patients with leukocytosis), by week 2 (n=7), 6 months (n=1), and 12 months (n=1) and was sustained in all but one patient. Sixteen patients (50%) had thrombocytosis at baseline defined as a platelet count > 400 x 10^9/l (median baseline platelet count 593 x 10^9/l, range 413-1010 x 10^9/l, in the 16 patients), which resolved in 14 of the patients by a median follow-up time of 1 month (range, 2 weeks-6 months), and was sustained ≥ 3 months including at 12 months of follow-up in eight patients. Baseline palpable splenomegaly was present in six patients (19%) with a median palpable spleen length at 7.5 cm (range, 2-21 cm), below the LCM. At 12 months of follow-up, the median palpable spleen length in these six patients was reduced to 4 cm (range, 0-9 cm), below the LCM, and had resolved completely in three of the patients. Splenomegaly by ultrasonography (longest diameter > 13 cm) was present in 19 patients (59%) at baseline with a median spleen length of 14.5 cm (range, 13.1-30 cm), and ultrasonographic normalization of spleen size was observed in eight patients by a median follow-up time of 6 months (range, 3-12 months).

II. Treatment efficacy in PROs (TSS) in PV

The MPN-SAF questionnaires were completed at all study visits by 27 patients (84%). Four patients missed completions at six study visits in total, including one patient who missed the completion at baseline (and 6 and 12 months), and one additional patient failed to complete the questionnaires at any study visit. The two latter patients were not included in the efficacy analysis in TSS (n=30). At 2 weeks, 11 patients (37%) had a > 50% reduction in TSS from baseline, and four patients (13%) had a median increase of 30% (range, 9-66%). All three patients, who achieved PR, were among the 11 patients with an early > 50% reduction in TSS (27% PR in early TSS responders versus 0% PR in early non-responders; *P*=.067).

Median TSS including range and 25 and 75 percentiles in PV patients at baseline (0 months), 2 weeks (0.5 month), 1 month, 3 months, 6 months, 9 months, and 12 months.

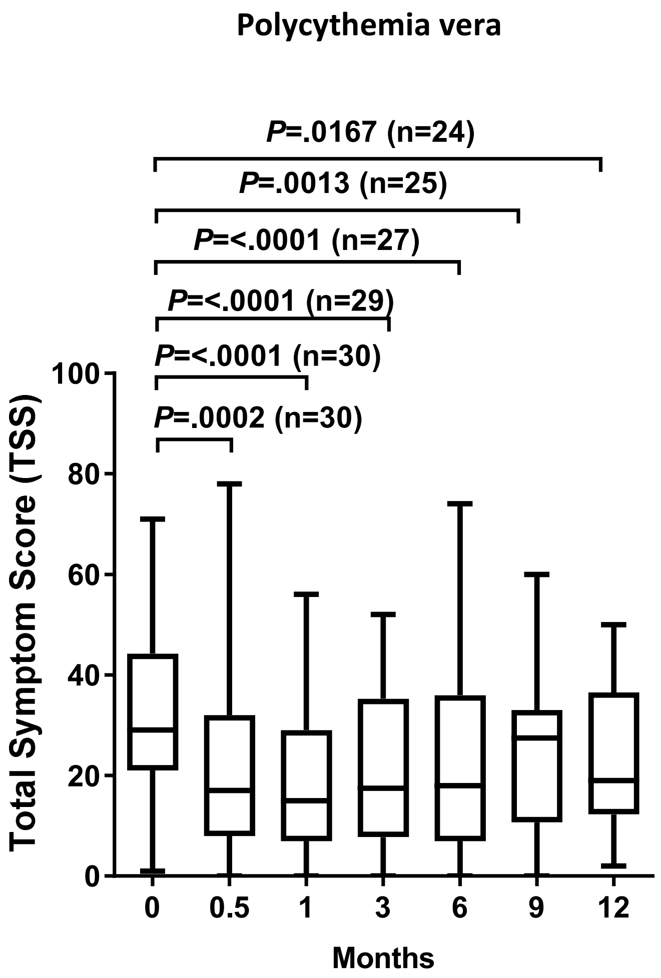

One missing value at 3 months, 6 months, and 9 months, respectively

Two patients were excluded due to missing values at 0 months

Supporting Information, Appendix S5b

A separate analysis of response in anemia or splenomegaly was not made in MF patients, since the remission definitions in MF include the category clinical improvement encompassing response in either of these parameters (or symptoms response) in patients not meeting the criteria for CR or PR. None of the patients fulfilled the criteria for the response category clinical improvement.

Treatment efficacy in PROs (TSS) in MF

The MPN-SAF questionnaires were completed at all study visits by 12 patients (67%). Five patients missed completions at six study visits in total, including one patient who missed the completion at baseline (and 2 weeks), and one additional patient failed to complete the questionnaires at any study visit. The two latter patients were not included in the efficacy analysis in TSS (n=16). At 2 weeks, three patients (19%; PMF, n=2; post-PV MF, n=1) had a > 50% reduction in TSS from baseline, and four patients (25%) had a median increase of 35% (range, 12-229%). Among the three patients with an early > 50% reduction in TSS, two patients (67%) achieved remission (complete and partial, respectively) compared to four of the remaining 13 patients (31%; *P*=.218; one patient achieving partial remission was omitted from the efficacy analysis in TSS due to failure to complete the questionnaires at baseline). The PMF patient with progressive disease had an increase in TSS between baseline and 2 weeks of follow-up of 12%.

Median TSS including range and 25 and 75 percentiles in MF patients at baseline (0 months), 2 weeks (0.5 month), 1 month, 3 months, 6 months, 9 months, and 12 months.

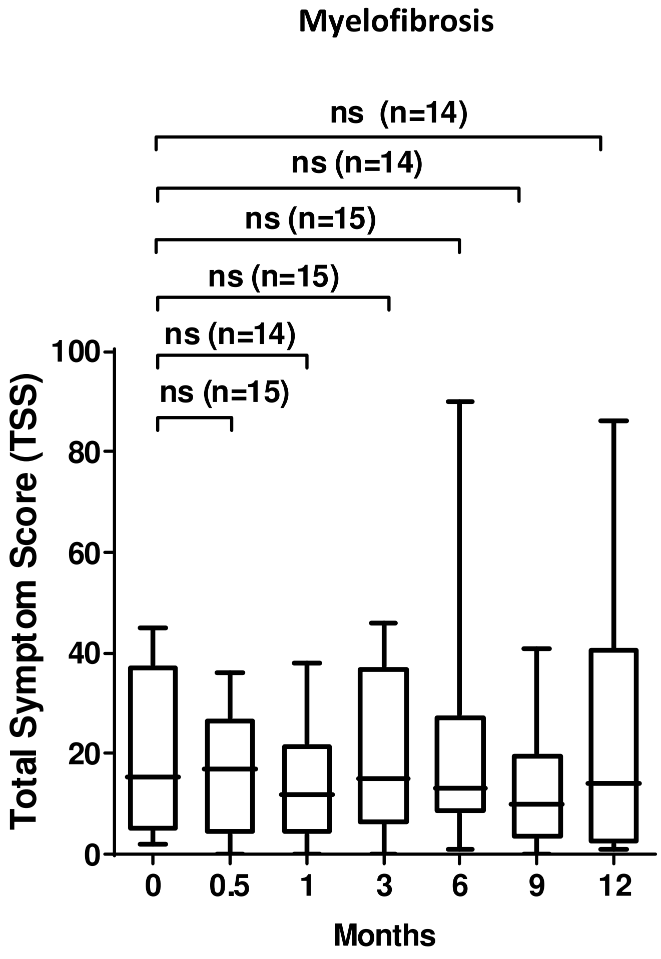

ns, non-significant (*P* value ≥ 0.05)

One missing value at 0.5 month; two missing values at 1 month; one missing value at 9 months

Two patients were excluded due to missing values at 0 months

Supporting Information, Appendix S6a

%V617F in PV patients at baseline, 3 months, 6 months, and 12 months of follow-up.

Patient number	*JAK2*V617F allele burden, %				Change in *JAK2*V617F allele burden between baseline and 12 months of follow-up/last study visit before discontinuation of study therapy (Δ%V617F/baseline %V617F), %
	Baseline	3 months	6 months	12 months	
1	43	38	42	*d/c*	↓ 2
2	61	17	*d/c*	*d/c*	↓ 72
3	87	89	84	89	↑ 2
4	88	78	65	60	↓ 32
5	38	37	35	39	↑ 3
6	16	14	9	15	↓ 6
7	5.9	2.9	1.9	*d/c*	↓ 68
8	1.8	0.74	0.74	0.73	↓ 59
9	19	17	31	14	↓ 26
10	59	20	28	8.6	↓ 85
11	78	56	42	42	↓ 46
12	56	33	50	50	↓ 11
13	26	20	19	18	↓ 31
14	53	50	44	52	↓ 2
15	78	80	*d/c*	*d/c*	↑ 3
16	13	12	15	8.3	↓ 36
17	51	41	32	27	↓ 47
18	81	54	52	63	↓ 22
19	15	10	8.6	*d/c*	↓ 43
20	14	7.1	8.5	5.7	↓ 59
21	93	79	77	50	↓ 46
22	41	29	32	21	↓ 49
23	97	92	95	88	↓ 9
24	30	25	16	4.5	↓ 85
25	90	75	81	77	↓ 14
26	18	24	13	12	↓ 33
27	39	28	29	26	↓ 33
28	92	73	74	83	↓ 10
29	75	62	56	17	↓ 77
30	84	54	*Missing value*	17	↓ 80
31	30	37	33	*d/c*	↑ 10
32	24	16	12	8.3	↓ 65

Abbreviations: JAK, Janus kinase; *d/c*, study medication discontinued

Supporting Information, Appendix S6b

Mutant allele burden in MF patients at baseline, 3 months, 6 months, and 12 months of follow-up.

Patient number	Mutant allele burden, %				Change in mutant allele burden between baseline and 12 months of follow-up/last study visit before discontinuation of study therapy (Δ%allele burden/baseline %allele burden), %
	Baseline	3 months	6 months	12 months	
***JAK2*-mutated patients**					
33	57	49	43	56	↓ 2
34*	41	24	18	42	↑ 2
35	97	94	93	95	↓ 2
36	0.1	0.1	0	0.08	↓ 20
37	66	63	63	49	↓ 26
38	49	51	55	*d/c*	↑ 12
39	11	7.8	8.5	4	↓ 64
40	61	32	27	15	↓ 75
41†	86	81	79	68	↓ 21
42	15	8.1	7.7	7.7	↓ 49
43	26	16	11	5.7	↓ 78
44	30	19	20	18	↓ 40
***CALR*-mutated patients**					
45	41	34	31	*d/c*	↓ 24
46	40	45	42	47	↑ 18
47	33	35	39	44	↑ 33
48	45	*d/c*	*d/c*	*d/c*	*n/a*

Abbreviations: %allele burden, mutant allele burden in %; JAK, Janus kinase; CALR, calreticulin; *d/c*, study medication discontinued; *n/a*, not applicable

*IFNα2 paused at 9 months of follow-up for > 3 months due to headache

†MF patient with progressive disease

The two triple-negative patients were excluded from the table

Supporting Information, Appendix S7

Non-hematologic adverse events, including serious adverse events, reported in ≥ 3 patients.

Adverse event	Total	Polycythemia vera		Myelofibrosis	
	All grades, n (n patients; % of all patients)	All grades, n (n patients; %)	Grade ≥ 3, n (n patients; %)	All grades, n (n patients; %)	Grade ≥ 3, n (n patients; %)
Arthralgia and/or myalgia	40 (24; 47%)	31 (17; 53%)		9 (7; 37%)	
Flu-like symptoms*	24 (16; 31%)	16 (11; 34%)		8 (5; 26%)	
Nausea	16 (13; 25%)	11 (9; 28%)		5 (4; 21%)	
Dizziness	15 (12; 24%)	7 (6; 19%)		8 (6; 32%)	
Fever	15 (7; 14%)	9 (5; 16%)	1 (1; 3%)	6 (2; 11%)	6 (2; 11%)
Headache	14 (9; 18%)	9 (6; 19%)		5 (3; 16%)	
Upper respiratory tract infection	13 (9; 18%)	6 (5; 16%)		7 (4; 21%)	
Injection site reactions*	12 (8; 16%)	8 (7; 22%)		4 (1; 5%)	
Bacterial infection, not otherwise specified	11 (10; 20%)	6 (6; 19%)	2 (2; 6%)	5 (4; 21%)	3 (2; 11%)
Neuropsychiatric symptoms, not otherwise specified	8 (8; 16%)	4 (4; 13%)		4 (4; 21%)	
Pneumonia	8 (8; 16%)	5 (5; 16%)	3 (3; 9%)	3 (3; 16%)	3 (3; 16%)
Dyspnea	8 (7; 14%)	5 (4; 13%)		3 (3; 16%)	
Neuropathy or related symptoms	8 (7; 14%)	5 (4; 13%)		3 (3; 16%)	
Arterial hypertension	7 (6; 12%)	7 (6; 19%)	5 (4; 13%)	0	
Heart palpitations	7 (6; 12%)	6 (5; 16%)		1 (1; 5%)	
Cough	7 (5; 10%)	2 (2; 6%)		5 (3; 16%)	
Fatigue	7 (5; 10%)	4 (3; 9%)		3 (2; 11%)	1 (1; 5%)
Dermatitis/Exanthema	7 (6; 12%)	2 (2; 6%)		5 (4; 21%)	
Elevated ALAT	6 (5; 10%)	5 (4; 13%)		1 (1; 5%)	
Angina pectoris	5 (5; 10%)	3 (3; 9%)		2 (2; 11%)	
Abdominal pain	5 (5; 10%)	1 (1; 3%)		4 (4; 21%)	1 (1; 5%)
Dyspepsia	5 (5; 10%)	2 (2; 6%)		3 (3; 16%)	
Diarrhea	5 (4; 8%)	2 (2; 6%)		3 (2; 11%)	
Weight gain	4 (4; 8%)	3 (3; 9%)		1 (1; 5%)	
Acne	4 (4; 8%)	1 (1; 3%)		3 (3; 16%)	
Dry skin	4 (3; 6%)	3 (2; 6%)		1 (1; 5%)	
Superficial hematomas	4 (3; 6%)	3 (2; 6%)		1 (1; 5%)	
Tinnitus	3 (3; 6%)	3 (3; 9%)		0	
Exhaustion	3 (3; 6%)	2 (2; 6%)		1 (1; 5%)	1 (1; 5%)
Visual disturbances	3 (3; 6%)	1 (1; 3%)		2 (2; 11%)	
Sinus bradycardia	3 (3; 6%)	1 (1; 3%)		2 (2; 11%)	
Dry mouth	3 (3; 6%)	2 (2; 6%)		1 (1; 5%)	
Depressed mood	3 (3; 6%)	2 (2; 6%)		1 (1; 5%)	
Other events of grade ≥ 3, each in < 3 patients	14 (14; 27%)	-	10 (10; 31%)	-	4 (4; 21%)

Abbreviation: ALAT, alanine transaminase

Total number of patients, n=51; PV patients, n=32; MF patients, n=19

*related to PEG-IFNα2 subcutaneous injection

Supporting Information, Appendix S8a

Dose modifications of study medication in PV patients

Twenty-two PV patients (69%) were reduced to a ruxolitinib twice-daily dose of ≤ 10 mg by a median follow-up time of 2 weeks (range, 0-6 months), and in 19 PV patients (59%), PEG-IFNα2 was at some point paused, and/or the dosing interval was extended.

Median daily dose of ruxolitinib, mg, and median weekly dose of PEG-IFNα2a, μg, including range and 25 and 75 percentiles in PV patients at baseline (0 months), 3 months, 6 months, 9 months, and 12 months.

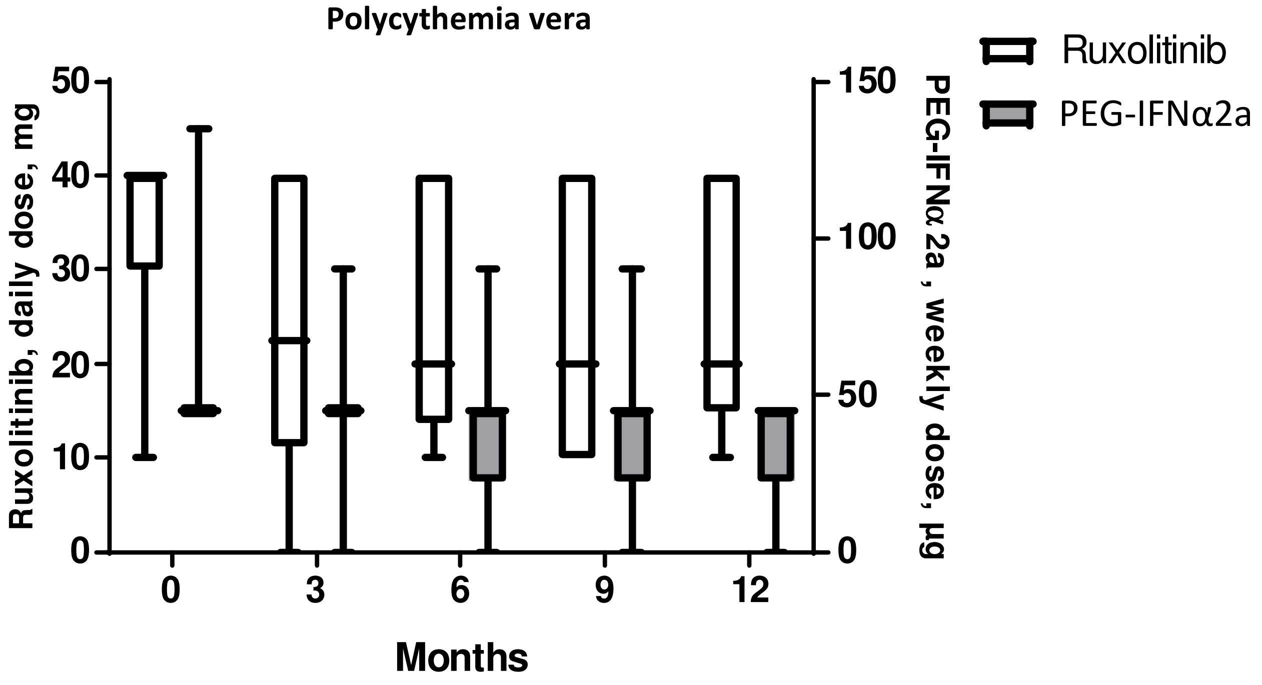

PEG-IFNα2b in PV patient(s)

One PV patient received PEG-IFNα2b 35 μg once weekly as initial therapy instead of PEG-IFNα2a. The patient was dose-reduced to PEG-IFNα2b 35 μg once every other week by 3 months and continued throughout follow-up. Hematologic toxicity, grade 1-2, was recorded in all three peripheral blood cell lines in addition to four non-hematologic AEs of grade 1-2 including one SAE (angina pectoris; considered unrelated to study therapy).

Supporting Information, Appendix S8b

Dose modifications of study medication in MF patients

Fourteen MF patients (78%) were reduced to a ruxolitinib twice-daily dose of ≤ 10 mg by a median follow-up time of 3 weeks (range, 0-7 months), and in 12 MF patients (67%), PEG-IFNα2 was at some point paused, and/or the dosing interval was extended.

Median daily dose of ruxolitinib, mg, and median weekly dose of PEG-IFNα2a, μg, including range and 25 and 75 percentiles in MF patients at baseline (0 months), 3 months, 6 months, 9 months, and 12 months.

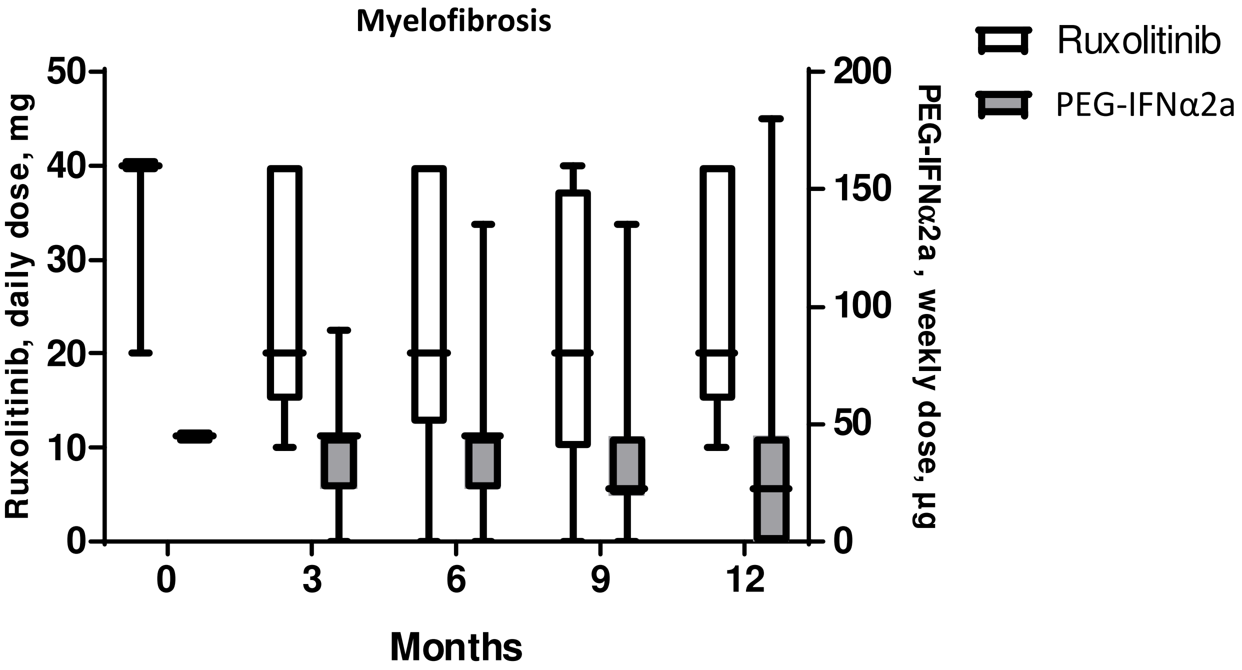

Abbreviation (both figures): PEG-IFNα2a; pegylated interferon-α2a

PEG-IFNα2b in MF patients

Two MF patients received PEG-IFNα2b 35 µg once weekly as initial therapy instead of PEG-IFNα2a. The two patients remained on the initial PEG-IFNα2b dose throughout follow-up. Anemia +/- thrombocytopenia, grade 1-2, was recorded in both patients in addition to a total of three non-hematologic AEs of grade 1-2 including one SAE (extremity pain; considered unrelated to study therapy).
